# Sensitive Detection of Foodborne Parasitic Larvae Using a Sieving–qPCR Workflow: Application to *Angiostrongylus cantonensis*

**DOI:** 10.1111/1750-3841.71260

**Published:** 2026-07-14

**Authors:** Jana Kačmaříková, Helena Stříbrná, Kateřina Janečková, Barbora Červená

**Affiliations:** ^1^ Department of Pathology and Parasitology University of Veterinary Sciences Brno Brno Czech Republic; ^2^ Institute of Vertebrate Biology Czech Academy of Sciences Brno Czech Republic

**Keywords:** *Angiostrongylus cantonensis*, food safety, foodborne pathogen, rat lungworm, sieving

## Abstract

Contaminated food and water represent potential infection sources of *Angiostrongylus cantonensis*, a parasitic nematode with significant zoonotic potential. Limited environmental surveillance may be due to the absence of simple and reliable screening methods. Different methods of *A. cantonensis* DNA detection were evaluated on lamb's lettuce (*Valerianella locusta*). First, three methods for concentrating larvae from water samples spiked with 1 and 10 third‐stage larvae (L3) were compared: simple sedimentation and two sieving techniques. Then, two DNA extraction modifications were assessed on spiked lettuce samples. Lastly, the detection limit was evaluated using lettuce samples spiked with 1, 5, 10, 20, and 50 L3. Method efficiency was assessed using a sensitive quantitative polymerase chain reaction (qPCR). The highest DNA yields were obtained using a sieving method with a direct sieve DNA extraction employing glass beads and chelating resin. Using this approach, the detection limit was determined to be a single L3 in 50 g of lamb's lettuce. Detection was successful from both the sieve‐retained material and the sedimented flow‐through. Sieving techniques provide a promising screening approach for detecting larval stages of parasites, such as *A. cantonensis* in vegetables. Combined with a sensitive qPCR, detection was consistently achieved at 1 L3 under controlled conditions. Detection of parasite DNA in environmental samples indicates a potential health risk and supports the applicability of this method for food safety monitoring, with possible adaptation to other vegetables.

## 1 Introduction

*Angiostrongylus cantonensis*, a zoonotic metastrongyloid nematode, exhibits a complex life cycle. The definitive hosts are rats, who harbor adult worms in their lung arteries and pass the first‐stage larvae (L1) in their feces. Various species of gastropods serve as intermediate hosts, who become infected primarily by ingesting rat feces with L1, and third‐stage larvae (L3) subsequently develop within their tissue. After ingestion by the rat definitive hosts, the larvae develop in the subadults in the central nervous system (CNS) and then migrate to the right heart cavities and pulmonary arteries. The L3 from snail tissue may be ingested by paratenic hosts, for example, freshwater shrimps, frogs, and lizards (Turck et al. 2022) in which the *A. cantonensis* larvae do not develop, but may become a source of infection for either the definitive hosts or accidental hosts. In accidental hosts, including humans, the larvae reach the CNS, and the subadults develop and burrow into neural tissue, where they eventually die, causing inflammatory lesions leading to eosinophilic meningitis (Graeff‐Teixeira et al. 2009), which may be potentially fatal (Bowden 1981; Cooke‐Yarborough et al. 1999; Lindo et al. 2004; Tangchai et al. 1967). Human infections are documented mostly after ingestion of raw or undercooked intermediate or paratenic hosts, unwashed produce contaminated by L3 (Banerjee et al. 2025; Hochberg et al. 2011), or from water sources where gastropods drowned and released L3 (Howe et al. 2019).

*A. cantonensis* was originally discovered in Southern China (Chen 1935), but its distribution is gradually expanding, largely driven by increasing globalization (Cowie et al. 2022). Molecular analyses have revealed high genetic diversity in *A. cantonensis* populations from Southeast Asia, whereas invaded regions typically show low diversity, often within a single lineage (Červená et al. 2019; Gómez‐Samblás et al. 2024; Tian et al. 2023). The parasite is now widespread in the Asia‐Pacific region, but reported also in Africa, the Americas, Australia, the Canary Islands, the Caribbean islands, Hawaii, and reports of newly invaded territories keep occurring with recent confirmations in continental Europe (Barratt et al. 2016; Galán‐Puchades et al. 2022; Pandian et al. 2025).

Various diagnostic approaches for *A. cantonensis* detection can be used, including microscopical and molecular methods. Microscopical methods can include the examination of adult worms recovered from the pulmonary arteries during necropsy of definitive hosts, the identification of L3 isolated from intermediate and paratenic host tissues using digestion methods, or the detection of L1 through larvoscopic examination of fecal pellets from definitive hosts. Although these techniques are relatively straightforward and cost‐effective, molecular confirmation is often recommended for accurate species identification, especially in areas where *A. cantonensis* co‐occurs with other *Angiostrongylus* spp., such as *Angiostrongylus malaysiensis*. Molecular methods primarily focus on the detection of *A. cantonensis* DNA using PCR‐based techniques in various materials, such as patient cerebrospinal fluid (CSF) (Ming et al. 2017), animal blood samples (Jarvi et al. 2024), tissues of intermediate or paratenic hosts (Anettová et al. 2022), and fecal samples (Fang et al. 2012). Loop‐mediated isothermal amplification (LAMP) has also been developed and successfully used to detect *A. cantonensis* in dog CSF (Baláž et al. 2023). These methods offer higher sensitivity and specificity, making them particularly valuable in early detection and epidemiological studies.

Relatively little attention has been given to environmental sources of infection, such as contaminated vegetables, soil, or water. Such material has widely been used for detection of soil‐transmitted helminths, including *Toxocara* spp., *Trichuris* spp., *Ancylostoma duodenale, Necator americanus*, and *Echinococcus* spp. (Amoah et al. 2017; Guggisberg et al. 2020; Manuel et al. 2024; Umhang et al. 2017). For helminth eggs, a combination of sieving and flotation is commonly used to detect the parasite stages from soil matrices, followed by molecular and/or microscopical detection, whereas sedimentation with Kato‐Katz or formol–ether concentration can be used for water samples (Aghaindum and Landry 2019). For food samples, water washing combined with sieving has been successfully employed to detect parasitic eggs, cysts, and oocysts (Berrouch et al. 2020), followed by immunomagnetic separation for *Giardia* and *Cryptosporidium* and microscopic evaluation (Robertson and Gjerde 2000), or using filters with varying mesh sizes to separate different types of organisms followed by PCR analysis (Guggisberg et al. 2020). A similar sieving approach has been adopted for lungworm L1 detection from fecal samples (Al‐Sabi et al. 2010).

To reduce the risk of human angiostrongyliasis originating from contaminated vegetables, a reliable screening methodology is essential. In this study, we aimed to develop a molecular approach for estimating *A. cantonensis* contamination, specifically focusing on scenarios where infective L3 may be present. L3 can contaminate produce intended for human consumption through various routes, including deposition via molluscan slime (Rollins et al. 2023; Šipková et al. 2024), accidental ingestion of small species of infected gastropods (Waugh et al. 2005), and paratenic hosts (Ash 1976), or irrigation and washing with water containing larvae released from drowned intermediate hosts (Howe et al. 2019). Although DNA‐based methods cannot differentiate between parasite life stages and may detect noninfective stages, such as L1, the presence of any *A. cantonensis* DNA nonetheless indicates environmental contamination and warrants caution. Such findings have important implications for food safety and public health. In this study, we propose a method for detecting *A. cantonensis* DNA in samples acquired by sieving water from vegetable washings followed by highly sensitive quantitative polymerase chain reaction (qPCR) that combines a laboratory‐feasible effective larval concentration step with a highly sensitive DNA detection assay, and with potential for routine screening of vegetable produce.

## 2 Materials and Methods

The experiment was conducted using a laboratory‐maintained strain of *A. cantonensis* originally obtained in Tenerife, Spain, in 2021, which has been circulating between rats (*Rattus norvegicus*, Wistar strain) and gastropods (*Lissachatina fulica*) in laboratory conditions since then. The experimental field isolate has been confirmed by sequencing of the adult nematodes’ gene for cytochrome c oxidase subunit I (COI), which identified the haplotype as a part of *A. cantonensis* clade 2 (Červená et al. 2019). Animals were kept in the animal facility of the Department of Pathology and Parasitology, University of Veterinary Sciences Brno, in accordance with national legislation (Approval of Ministry of Education, Youth and Sports of the Czech Republic no. MSMT‐20138/2023‐3).

### 2.1 Larval Isolation

Larvae used in this study were obtained from gastropod tissue using an artificial digestion method. The gastropods were first euthanized by decapitation, the muscles were then cut into smaller pieces and placed into digestion fluid consisting of 3 g of pepsin and 28 mL of 25% HCl in a total volume of 1 L of distilled water. The tissues were left stirring at 37°C until fully digested. The mixture was then centrifuged, sediment was examined under a light microscope, and individual living third‐stage larvae (L3) were transferred to separate microcentrifuge tubes using a micropipette. To minimize the risk of accidental infection, the larvae were subsequently killed by exposure to the temperature of 71°C for 60 s (Alicata 1967).

### 2.2 *Angiostrongylus cantonensis* DNA Detection

*A. cantonensis* DNA was detected using a highly sensitive, species‐specific qPCR assay (Sears et al. 2021). The assay was performed in a 20 µL reaction volume, containing 10 µL of 2× MasterMix (PrimeTime Gene Expression Master, Integrated DNA Technologies, Inc., Coralville, IA, USA), 6.2 µL of PCR water, 0.8 µL of each 10 µmol/L primer (F: 5′‐AAACTGTTGCTTTCGAAGCTATG‐3′, R: 5′‐GCGCAAATCTGACGTTCTTG‐3′), 0.2 µL of 10 µmol/L probe (PrimeTime Eco Probe 5′ 6‐FAM/ZEN/3′ IBFQ, /56‐FAM/ACA TGA AAC/ZEN/ACC TCA AAT GTG CTTCGA/3IABkFQ/), and 2 µL of DNA template. Thermocycling was performed on the Roche LightCycler 480 platform with the following cycling conditions: 40 cycles at 95°C for 20 s, 40°C for 1 s, and 60°C for 20 s. All samples were analyzed in duplicates with a cut‐off for positive sample set on 35 cycles. *C*_p_ values were determined by default threshold setting in LightCycler 480 Software (Roche Diagnostics, Switzerland). As a positive control, DNA was extracted from 10 *A. cantonensis* L3 using DNeasy Blood & Tissue (Qiagen, Germany) and subsequently diluted 10×. For quantification using larval genomic equivalents, an external calibration curve was created by serial dilution of DNA extracted from 10 *A. cantonensis* larvae, resulting in genomic equivalents of 10, 1, 0.1, 0.01, and 0.001 L3. Quantification was performed in duplicate for each sample, and the resulting values were averaged. The mean genomic equivalents (MGE) were calculated as the average of independent technical runs.

### 2.3 Larval Concentration Optimization

**FIGURE 1:**
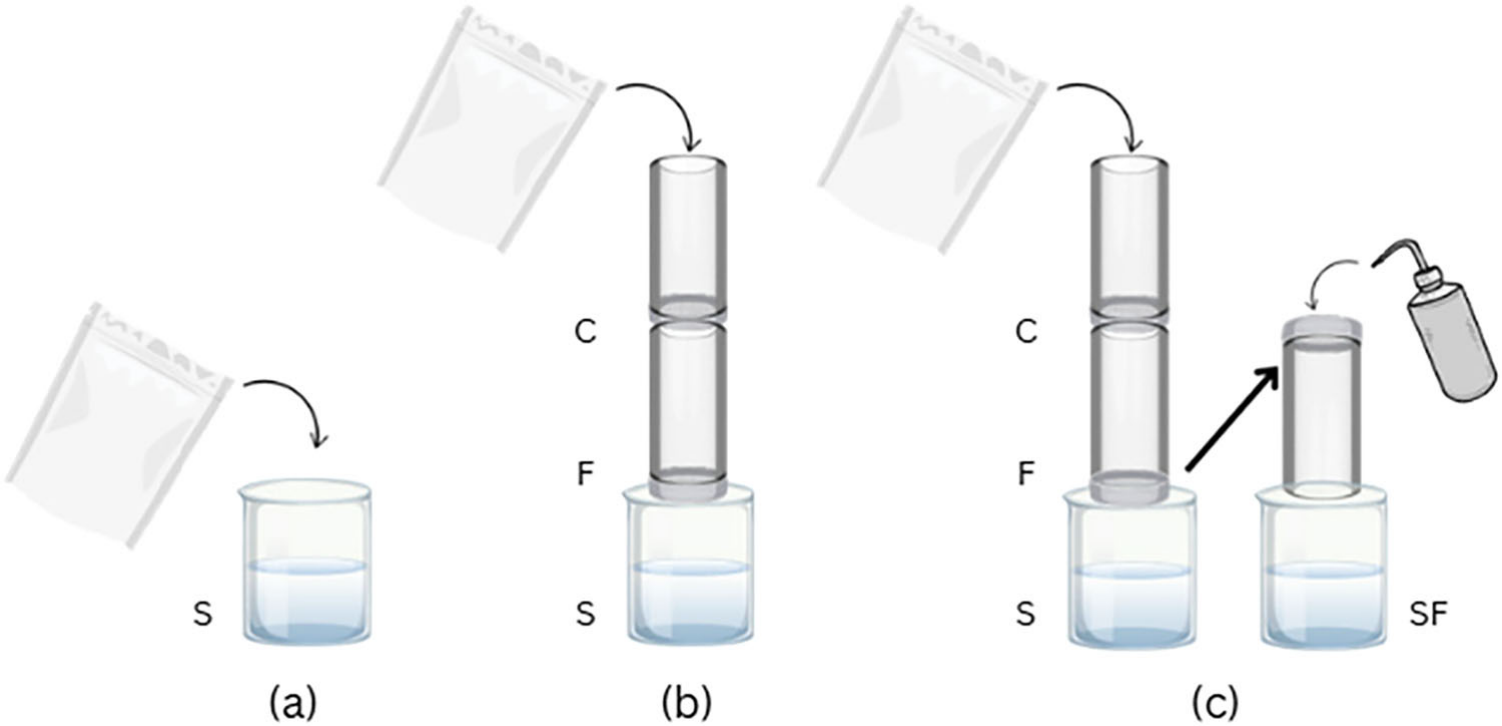
Larval concentration methods: (a) simple sedimentation method; (b) sieving method with sieve extraction; and (c) sieving method with sieve flushing. C, coarse sieve; F, fine sieve; S, sediment; SF, sieve flushing.

In the first step, several methods were tested to determine the most accurate and simple way for detection and concentration of *A. cantonensis* L3 larvae. Two aliquots of tap water (400 mL) spiked with 1 and 10 L3 were used for testing. Tested methods included (a) simple sedimentation, (b) sieving with direct sieve extraction, and (c) sieving with subsequent sieve flushing (Figure 1).

For the simple sedimentation method, the spiked water was poured into a 500 mL glass beaker and left to sediment for 30 min. The supernatant was then discarded, and 50 mL of the sediment was transferred into a centrifuge tube and centrifuged (2 min, 2000 rpm); then, after decanting the supernatant, 1.5 mL of the sediment was transferred to a microcentrifuge tube by a Pasteur pipette and used for DNA extraction after another centrifugation and removing the supernatant, until 0.2 mL of the sediment was left.

For the sieving methods, an apparatus consisting of two sieves with the mesh widths 400 µm × 200 µm and 21 µm × 21 µm (Lanz‐Anliker AG, Switzerland) was used. Each sieve was attached to a modified 50 mL Falcon tube with perforated lid and the bottom part removed. The Falcon tubes with sieves were connected using funnels constructed from single‐use plastic cups. The first coarser sieve was intended to retain dirt and plant debris, whereas the finer second sieve aimed to capture larvae on the basis of their dimensions, as the L3 larvae measure approximately 474 µm in length and 26 µm in width (Ash 1970). To prevent contamination, each set of Falcon tubes with sieves was used only once. The flow‐through of the 400 mL of spiked water was collected in a 500 mL glass beaker, and the sediment was processed as described above for the simple sedimentation method. This accounted for the possibility of detecting DNA from damaged larval tissue and potential eDNA in the real setup. Three subsamples were used for DNA extraction: Sample C from the first sieve (coarse, 400 µm × 200 µm), Sample F from the second sieve (fine, 21 µm × 21 µm), and Sample S from the sediment obtained from the flow‐through. For the direct sieve DNA extraction, the sieves were pushed from the lid directly into 2 mL microcentrifuge tubes using sterile Pasteur pipettes. In the case of sieve flushing, the modified Falcon tubes were reversed, and the material caught on the second sieve was washed into a centrifuge tube using 50 mL of water from a wash bottle, centrifuged (1 min, 1500 rpm), and 0.1 mL of the sediment used for DNA extraction, adding one more subsample for DNA extraction (Sample SF—sieve flushing). The flushed fine sieve was also used for DNA extraction (Sample F) to verify if all larvae were flushed or if any remained on the sieve.

DNA from all these samples was extracted by DNeasy Blood & Tissue kit (Qiagen, Germany) with overnight tissue lysis and elution performed in two steps, using 50 µL of the elution buffer in each to obtain the total volume of 100 µL. The samples were then analyzed by qPCR. Each concentration method was repeated five times for each aliquot (1 and 10 L3), and qPCR was performed in duplicates.

### 2.4 DNA Extraction Optimization

Due to the possible presence of DNA inhibitors in plant tissue, two different methods of DNA extraction were tested, each in five replicates. For this step, 50 g of lamb's lettuce (*Valerianella locusta*) were mixed with 50 mL of tap water. Then, 0.5 mL of the resulting mixture was transferred to a microcentrifuge tube, and 10 L3 larvae were added directly to the tube. DNA was then extracted using either (1) DNeasy Blood & Tissue kit (Qiagen, Germany) with the tissue lysis time prolonged to 6 h or (2) modified Blood & Tissue (Qiagen, Germany). The modification involved adding 0.1 mL of 0.5 mm glass beads (BioSpec, USA) after the tissue lysis step, followed by 30 min of vortexing at 2700 rpm using a Vortex Genie 2 (Scientific Industries, USA) with a Horizontal Microtube Holder SI‐H524. The sample was then centrifuged, and the supernatant transferred to a new microcentrifuge tube prior to the addition of Buffer AL. After the subsequent cell lysis step, 0.1 mL of Chelex 50 solution (Bio‐Rad, USA) was added, followed by another 30‐min vortexing. Extracted DNA was then used for qPCR analysis performed in duplicates of DNA aliquots.

### 2.5 Detection Limit Determination

**FIGURE 2:**
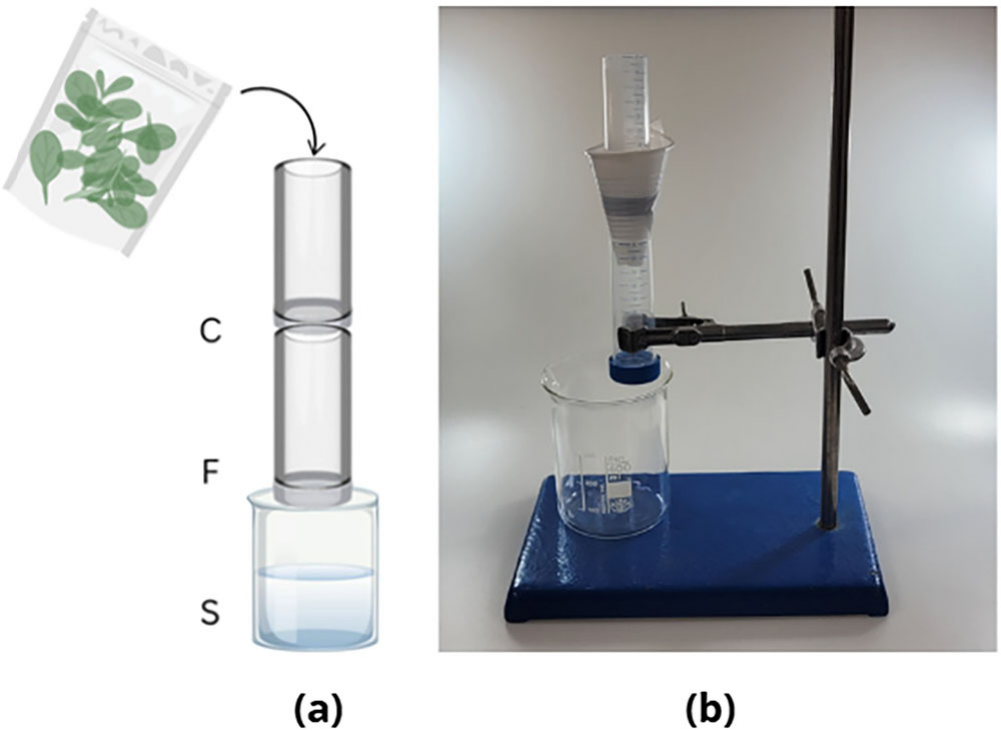
Final larval concentration method for detection limit determination: (a) schematic visualization and (b) photograph of the method.

For detection limit determination, lamb's lettuce (*V. locusta*) spiked with a known number of L3 was used. To simulate a portion a single person would consume in a meal, 50 g of lamb's lettuce was spiked with 1, 5, 10, 20, and 50 L3. The salad samples were subsequently put in individual 1000 mL ziplock bags with 400 mL of tap water added. The bag was placed onto an automatic stirrer (1 min, 500 rpm) to stimulate the release of L3 into water. For L3 concentration from the water medium, a sieving apparatus containing a sieve with a mesh width of 400 µm × 200 µm (Sample C) and another sieve with a mesh width of 21 µm × 21 µm (Sample F) was used (Figure 2). If a sieve got blocked by the debris, a clean Pasteur pipette was used to stir the liquid to enable passing through the sieve. The flow‐through was captured in a glass beaker and left to sediment for 30 min; then the supernatant was discarded, and 50 mL of sediment was transferred into a tube and centrifuged (2 min, 2000 rpm). Utilizing a clean Pasteur pipette, 1.5 mL of the sediment was transferred to a microcentrifuge tube and used for DNA extraction (Sample S).

The DNA extraction was conducted on Sample F (fine sieve) and Sample S (sediment from the flow‐through) using the modified Blood & Tissue (Qiagen, Germany) method as described above. Sample S was first centrifuged, and then the supernatant was discarded to leave up to 0.2 mL of final sediment. Extracted DNA was then used for the qPCR analysis, and samples were analyzed in duplicates.

### 2.6 Statistical Evaluation

Data were analyzed using GraphPad Prism version 10.6.0 (GraphPad Software, USA). To compare *C*_p_ values among different sample processing methods, the Kruskal–Wallis test followed by Dunn's post hoc test with multiple comparison correction was applied. Differences between the two DNA extraction protocols were evaluated using an unpaired two‐tailed *t*‐test. Linear regression analysis was performed to assess the relationship between the number of input larvae and *C*_p_ values, with regression slopes calculated. A *p* value <0.05 was considered statistically significant.

### 2.7 Artificial Intelligence

Artificial intelligence tools (ChatGPT, OpenAI) were used for language editing and refinement of the text and did not generate any scientific content, data, or conclusions.

## 3 Results

During the larval concentration optimization, the efficiency and laboratory‐feasibility were evaluated and compared among three different methods, each repeated five times. The results are presented as mean *C*_p_ values with the standard deviations (SD) and as mean genomic equivalents (MGE) across replicates in Table 1.

**TABLE 1:** Comparison of larval concentration methods for quantitative polymerase chain reaction (qPCR) detection of *Angiostrongylus cantonensis* in experimentally spiked water samples.

		Sedimentation		Sieving—extraction		Sieving—flushing	
	Sample	*C*_p_ ± SD	MGE	*C*_p_ ± SD	MGE	*C*_p_ ± SD	MGE
**10 L3**	C	N/A	N/A	25.86 ± 2.592	0.0038	24.21 ± 5.135	0.1479
	F	N/A	N/A	11.41 ± 0.348	9.9450	17.62 ± 3.637	0.8858
	S	21.33 ± 7.870	1.9004	26.16 ± 1.655	0.0022	N/A	N/A
	SF	N/A	N/A	N/A	N/A	15.56 ± 4.491	2.3271
**1 L3**	C	N/A	N/A	27.44 ± 1.243	0.0006	27.31 ± 0.999	0.0004
	F	N/A	N/A	19.11 ± 5.404	0.6598	26.35 ± 1.081	0.0012
	S	26.96 ± 1.394	0.0008	27.90 ± 1.466	0.0003	N/A	N/A
	SF	N/A	N/A	N/A	N/A	22.79 ± 4.023	0.1348

*Note: C*_p_ of positive control (10 L3) = 11.145.

Abbreviations: C, coarse sieve; F, fine sieve; MGE, mean genomic equivalent; N/A, not applicable; S, sediment; SF, sieve flushing.

*A. cantonensis* DNA was successfully detected in all analyzed samples. No amplification was observed in negative controls. However, there were significant differences in efficiency among the methods for both larval counts used for spiking (Kruskal–Wallis *p *= 0.015 for 1 L3*; p *= 0.0018 for 10 L3). Dunn's post hoc test revealed a significant difference between sedimentation versus direct sieve extraction with both 1 L3 *(p *= 0.033) and 10 L3 *(p *= 0.011) and between direct sieve extraction versus sieve flushing *(p *= 0.049) for 10 L3 used. In the sedimentation method, the detected *C*_p_ values were higher than in other methods, indicating lower DNA yield. Sieving methods showed higher yield of DNA, but the two approaches also differed. The *A. cantonensis* DNA was detected in both crucial samples—Sample F (fine sieve) in direct DNA extraction from the sieves and Sample SF (sedimentation from sieve flushing) in the sieve flushing method. However, the *C*_p_ values were much lower, and genomic equivalents were much closer to the used number of L3 for the direct DNA extraction method. Additionally, manipulation with the sieves was easier for the direct extraction as well. Therefore, the sieving method with the DNA extraction directly from the sieves was identified as the most suitable approach for our objectives.

Comparison of the two DNA extraction protocols also showed differences in performance. The Blood & Tissue (BT) protocol resulted in significantly higher *C*_p_ values than the modified Blood & Tissue (ABT) method (unpaired *t*‐test, two‐tailed; *n* = 5 per group; *t*(8) = 5.22, *p = *0.0008; mean ± SD: ABT = 12.85, BT = 15.33; difference = 2.48 ± 0.48 SEM, 95% CI 1.39–3.58; *η* ^2^ = 0.77). Although the mean *C*_p_ value of the DNeasy Blood & Tissue kit (Qiagen, Germany) protocol from five repetitions in duplicates was 15.33 (14.02–16.47), the mean *C*_p_ value in the modified protocol was 12.85 (12.13–13.3) with the positive control (DNA extracted from a suspension of 10 L3 in water) having the *C*_p_ 11.15. Thus, the modified DNA extraction protocol was further used in the study to estimate the detection limit.

During the last phase, the detection limit of the sieving method with the direct sieve extraction followed by the DNA extraction according to the modified protocol was determined. All tested samples, comprising five replicates for five different L3 concentrations for the fine sieve (Sample F) and the flow‐through sediment (Sample S), yielded positive results with *C*_p_ values and larval genomic equivalents varying among the L3 concentrations (Table 2). No amplification was observed in negative controls.

**TABLE 2:** Detection limit of the optimized sieving and quantitative polymerase chain reaction (qPCR) protocol for *Angiostrongylus cantonensis* L3 in experimentally spiked lettuce samples.

	Fine sieve (Sample F)			Sedimentation (Sample S)		
*n* L3	Mean *C*_p_ ± SD	MGE	Estimated recovery (%)	Mean *C*_p_ ± SD	MGE	Estimated recovery (%)
50	10.84 ± 0.706	12.6430	25.286	25.30 ± 6.828	0.2265	0.453
20	13.14 ± 0.790	2.6180	13.090	22.40 ± 4.195	0.0153	0.076
10	13.09 ± 0.938	2.8208	28.208	27.71 ± 2.075	0.0003	0.003
5	15.78 ± 1.420	0.5862	11.723	24.13 ± 7.796	0.1677	3.353
1	18.19 ± 0.817	0.0791	7.908	28.91 ± 3.142	0.0004	0.035

*Note: C*_p_ of positive control (10 L3) = 11.03; MGE = 10.0650.

Abbreviation: MGE, mean genomic equivalent.

A strong linear relationship was observed between the input larval number and *C*_p_ values in sieve samples (*R* ^2^ = 0.8597, slope = −4.318*, p* < 0.0001), suggesting a relationship between input larvae and *C*_p_ values under experimental conditions. Mean *C*_p_ values decreased with increasing larval input, with coefficients of variation ranging from 4.491% at 1 L3 to 6.516% at 50 L3. Sediment samples did not show significant linear correlation (*R* ^2^ = 0.07004, slope = −2.429, *p *= 0.2011) with overall lower DNA yield indicated by high *C*_p_ values. All replicates were positive down to 1 L3, indicating a practical detection limit of 1 larva under experimental conditions for both fine sieve and sediment samples.

## 4 Discussion

The emerging nature of *A. cantonensis* highlights the need for further screening possibilities not only in human and animal clinical samples but also in environmental and food samples. Food safety is crucial in the epidemiology of orally transmitted parasitic diseases, as early detection of the parasite contamination can prevent human infections.

In the present study, we aimed to develop a methodology for *A. cantonensis* screening in vegetables as a part of food safety measures by testing different methods of larval concentration and DNA extraction best suited to such sample types. Although we used an approximate single portion of lamb's lettuce, this methodology can be easily adopted for other types of vegetables and fruits as well, provided the sample fits into a 1000 mL ziplock bag and can be rinsed in 400 mL of water.

The tested methods included simple sedimentation, representing an approach requiring minimal equipment, and two sieving‐based methods. In the sedimentation method, the volume of the water medium was limited by the capacity of the sedimentation containers. When applied to water suspensions derived from vegetable washings, this approach may result in disproportionately large sample volumes with high amounts of debris due to the inability to effectively remove the dirt and plant material. For this reason, we proceeded with the sieving methods. Sieving with a direct sieve extraction proved to be the most successful during the concentration method optimization phase given its easier execution with minimal risk of contamination or DNA loss compared to the sieving method with sieve flushing, which required manually reversing the sieve, flushing the larvae into a tube, and performing an additional sedimentation step. This made the latter approach more labor‐intensive and prone to potential handling errors. The sieving methods also allow for the potential concentration of higher water volumes compared to sedimentation and may also be applied to larger volumes of water, such as in hydroponic or aquaponic farming where snails could have access, or aquatic snail farming. In cases where the presence of other parasites is suspected, a microscopic examination of the sieve can be performed (Guggisberg et al. 2020); however, this approach was not evaluated in the present study. Although the direct sieve DNA extraction allows for the detection of *A. cantonensis* without prior microscopic evaluation, it does not distinguish between developmental stages. As a result, both infective L3 and noninfective L1 may be detected. Nevertheless, the detection of *A. cantonensis* DNA in environmental or food samples should be regarded as a significant public health concern, as it confirms the parasite's presence in the environment and the potential for contamination of food intended for human consumption by infective L3. Given that even a very small number of infective larvae may be sufficient to cause disease (Rosen et al. 1967) depending on host immune status and larval infectivity, monitoring environmental sources is crucial to prevent human exposure.

Although other larval recovery methods, such as the Baermann technique followed by microscopic analysis, are useful for detecting motile larvae in heavily contaminated samples that are more likely to pose an infection risk, their sensitivity may be limited in environmental matrices with low parasite burdens, which still may represent a potential health risk. Moreover, in heterogeneous environmental samples, contamination may vary within a single production lot, with some subsamples containing higher larval burdens than others. Molecular screening approaches may therefore improve the likelihood of detecting contamination and confirming circulation of the parasite life cycle in the environment.

Different DNA extraction methods were tested to account for the possible presence of DNA inhibitors in the plant tissue retained on the sieve among the larvae. The best results were achieved using the Blood & Tissue extraction kit (Qiagen, Germany) together with Chelex 50 solution (Bio‐Rad, USA) designed to bind metal ions, inhibit nuclease activity, and remove PCR inhibitors, and with bead‐based mechanical disruption of the larval sheath of *A. cantonensis* L3. The modification improved the performance of the kit for our sample types without significant increase in cost or complexity. DNA extraction from L3 larvae can be challenging due to their resilient sheath. Prolonging tissue lysis with this kit for at least 6 h, instead of the standard 1 h recommended by the manufacturer, has been reported as an efficient approach for *A. cantonensis* L3, particularly when extracted from host tissues (Anettová et al. 2024). In samples containing plant‐derived inhibitors, extending the lysis time alone was not sufficient. Mechanical disruption with glass beads, combined with Chelex 50 solution treatment, provided a more effective alternative while also requiring a shorter extraction time.

The chosen DNA extraction method produced higher *C*_p_ values corresponding to lower DNA concentrations compared to the positive control. However, the positive control contained suspension of larvae in water and lacked any plant material that could introduce potential PCR inhibitors, which likely contributed to the higher DNA yield observed. The quantified genomic equivalents of the analyzed samples should be thus regarded as conservative estimates, because the true parasite load in tested samples is likely underestimated.

Nonetheless, the final selected methodology showed high sensitivity during the last phase of experimental trials—the detection limit determination, revealing positive results not only on the fine sieve, where the highest DNA concentrations are anticipated, but also in the sediment of the flow‐through. The amount of detected DNA in the sediment may be caused by the shedding of remnants of old cuticular layers from previous larval molts (Zeng et al. 2013). The highly sensitive AcanR3990 protocol used in this study is able to detect even 1 fg of *A. cantonensis* DNA in the sample (Sears et al. 2021), corresponding to approximately 1/100,000 of an L3 and, therefore, enables detection even when intact larvae are absent from the flow‐through. For future applications, it would be ideal to analyze both the fine sieve and the sediment from each sample, but a more cost‐effective way would be to prioritize the fine sieves, where larvae are most likely to be concentrated, while adopting a pooled‐sample approach for the flow‐through samples to reduce the number of assays required.

## 5 Limitations

It is also necessary to acknowledge limitations of the current study. All experiments represent technical replicates performed under controlled laboratory conditions using a single larval source. Therefore, variability associated with biological samples, environmental contamination, and inter‐sample heterogeneity is not captured. The use of direct larval spiking represents an idealized contamination model and likely results in higher recovery efficiency compared to natural contamination scenarios, which were not assessed in this study. Potential PCR inhibition was not directly evaluated using internal amplification controls, which may affect quantitative accuracy. Finally, although DNA detection indicates environmental contamination and potential exposure pathways, it does not confirm the presence of viable or infective larvae. Despite these limitations, the methods were systematically evaluated under controlled conditions and demonstrated consistent performance, suggesting their suitability for application to natural samples, although further validation is required to determine their performance under field conditions. Another important consideration is the representativeness of environmental sampling, as contamination of vegetable batches is likely to be heterogeneous and subsample analysis may not reflect contamination of an entire production lot. Therefore, the presented methodology is best suited for screening and surveillance purposes, providing a practical framework that may be adapted for targeted investigations or routine monitoring in high‐risk settings.

## 6 Conclusion

Using washing of vegetables followed by sieving, we were able to detect a single L3 in 50 g of lamb's lettuce (*V. locusta*), demonstrating a highly sensitive screening method. Focusing on food safety, this approach could be alternatively used on various types of vegetables and fruits where gastropod contamination is possible, including aquaponic or aquatic snail farming.

## Author Contributions

**Jana Kačmaříková**: writing – original draft, methodology, software, data curation, validation, conceptualization, investigation, formal analysis, visualization, project administration. **Helena Stříbrná**: conceptualization, supervision, funding acquisition. **Kateřina Janečková**: methodology, investigation. **Barbora Červená**: conceptualization, methodology, supervision, writing – review and editing, funding acquisition, project administration, resources.

## Funding

This research was supported by Internal Grant Agency of the University of Veterinary Sciences Brno (IGA VETUNI 113/2024/FVL).

## Ethics Statement

We hereby declare that all animal handling and experimental procedures adhered to ethical standards and were conducted under the approval of Ministry of Education, Youth and Sports of the Czech Republic no. MSMT‐20138/2023‐3.

## Conflicts of Interest

The authors declare no conflicts of interest.

## Data Availability

Additional data are available from the corresponding author upon request.
